# Metatranscriptomic Analysis Reveals Functional Features of the Gastrointestinal Microbiota in Tibetan Pigs

**DOI:** 10.3390/ani16182861

**Published:** 2026-09-11

**Authors:** Zhiyuan Xu, Xuanlin Sun, Yan Liang, Xuan Tao, Bo Ran, Pengliang Liu, Yiren Gu

**Affiliations:** 1 College of Animal Science and Veterinary Medicine, Southwest Minzu University, Chengdu 610041, China; 2 Animal Genetic Breeding and Reproduction Key Laboratory of Sichuan Province, Sichuan Animal Science Academy, Chengdu 610066, China

**Keywords:** Tibetan pig, black pig, metatranscriptome, gastrointestinal tract, microbial community

## Abstract

Tibetan pigs exhibit remarkable adaptability to hypoxic high-altitude conditions. However, metatranscriptomics has not been used to investigate the transcriptional activity and functional responses of the gastrointestinal microbiota as part of their adaptation to high altitude. We collected 57 samples of the contents of the stomach, jejunum, cecum, colon, and rectum of six high-altitude Tibetan pigs (n = 6, elevation 3750 m) and six low-altitude black pigs (n = 6, elevation 500 m) for metatranscriptomic analysis. We identified 79 differentially abundant bacterial genera and 69 differentially abundant bacterial species. In particular, *Lactobacillus crispatus* and *Lactobacillus delbrueckii* were significantly more abundant in the gastrointestinal microbiota of the high-altitude Tibetan pigs than in that of the low-altitude black pigs. Functional analysis revealed differences in transcripts associated with energy, carbohydrate, and amino acid metabolism. The microbial correlation network of Tibetan pigs contained more nodes and edges than that of black pigs. Consistent with this, within the CAZyme database, the CBM32 family of carbohydrate-binding modules and GH27 glycosidases were significantly enriched in the Tibetan pig samples, suggesting that these enzymes may play a crucial role in energy acquisition and carbon source metabolism in these pigs. These findings provide insights into the gastrointestinal microbial characteristics of Tibetan pigs and black pigs. In summary, these changes may enhance the stability of the gut ecosystem of Tibetan pigs and improve their energy utilization in nutrient-deficient high-altitude environments. These findings suggest that the gut microbiota may contribute to metabolic regulation in Tibetan pigs at high altitude and provide important clues regarding the gastrointestinal adaptations of high-altitude animals.

## Simple Summary

Elucidation of the role of the gut microbiota in adaptation to high-altitude environments is crucial to broaden understanding of the mechanisms whereby animals adapt to high altitude. We collected 57 samples of the contents of the stomach, jejunum, cecum, colon, and rectum from six Tibetan pigs living at high altitude and six black pigs living at low altitude and performed metatranscriptomic sequencing. This revealed that *Lactobacillus*, *Clostridioides*, and *Prevotella* were the most abundant genera in the gastrointestinal microbiota of both groups. *Lactobacillus crispatus* and *Lactobacillus delbrueckii* were significantly enriched in the gastrointestinal microbiota of the Tibetan pigs. The former is associated with gut health maintenance, and the latter is linked to appropriate lipid metabolism. Metabolic pathway analysis of the microbial communities revealed that the gut microbiota of the Tibetan pigs was significantly associated with energy metabolism and amino acid metabolism pathways, suggesting that these pathways may play a key role in metabolic regulation at high altitudes. In addition, network analysis indicated that the gut microbiota of the Tibetan pigs was more complex. These results characterize differences between the gastrointestinal microbial profiles of the two pig populations. These findings may advance understanding of the adaptations of the gastrointestinal tract to high-altitude environments.

## 1. Introduction

The Qinghai–Tibet Plateau is the highest plateau on Earth, with a mean elevation of approximately 4000 m [1]. It features extreme environmental conditions, including low oxygen levels, frigid temperatures, and intense ultraviolet radiation. Survival in such high-altitude environments poses significant physiological challenges for mammals [2]. Tibetan pigs (TPs) are one of the livestock populations native to this area and are primarily found in the agro-pastoral transition zone of the Qinghai–Tibet Plateau [3]. Tibetan pigs exhibit several morphological and physiological characteristics associated with high-altitude adaptation, including dense bristles, thick alveolar septa, and a dense capillary network [4,5]. These characteristics make Tibetan pigs a valuable genetic resource for investigating the mechanisms underlying adaptation to high-altitude environments.

The primary functions of the gastrointestinal tract are to digest food and absorb nutrients [6]. The stomach is responsible for the initial breakdown of food, the small intestine is primarily responsible for the absorption of nutrients, and the large intestine is primarily responsible for the absorption of water and electrolytes [7,8]. The gut microbiota of pigs influences their energy metabolism and immune responses by fermenting dietary fiber to produce short-chain fatty acids (SCFAs), synthesizing vitamins, and regulating the levels of neurotransmitter precursors, thereby playing crucial roles in the growth and immunity of pigs [9,10]. The results of recent studies suggest that the gut microbiota may also play an important role in the adaptation of mammals to high-altitude environments [11,12]. For example, the relative abundance of *Prevotella* in the gut microbiota of populations living in high-altitude regions is significantly higher than that of populations in low-altitude regions. This bacterial genus provides energy to the host by breaking down complex polysaccharides [13]. Another study showed that the gut microbiota of Tibetan antelopes possesses a greater capacity to ferment fiber, and the expression of genes related to energy metabolism is also significantly higher than in animals in low-altitude regions [14]. Therefore, characterization of the differences in the gastrointestinal microbiota of high-altitude and low-altitude pigs should not only expand understanding of the role of the gut microbiota in the adaptation of mammals to high altitude but also provide a new avenue for research on such adaptations.

In contrast to metagenomic sequencing, metatranscriptomic sequencing not only reveals the composition and structure of microbial communities but also captures the genes expressed by the gut microbiota, thereby reflecting the metabolic dynamics and functional activity of microorganisms under specific conditions, such as environmental changes, pathological states, or dietary factors [15,16,17]. Previous studies have demonstrated that, under hypoxic conditions at high altitudes, the gut microbiota of cattle and Tibetan sheep facilitate the adaptation of their hosts by modulating energy metabolism, promoting hematopoiesis, and enhancing antioxidant defenses [18,19]. However, systematic metatranscriptomic investigations of the gastrointestinal microbiota in Tibetan pigs inhabiting high-altitude environments remain scarce.

In the present study, we performed metatranscriptomic sequencing of samples collected from five gastrointestinal segments (the stomach, jejunum, cecum, colon, and rectum) of the Tibetan pigs (TPs) and black pigs (BPs) living at high and low altitudes, respectively. We compared the composition of the gut microbiota of these two groups and identified functional differences through KEGG pathway enrichment analysis. Additionally, we constructed bacterial correlation networks for the two groups and analyzed the expression of CAZyme genes in the gut microbiota. This study provides a comparative metatranscriptomic characterization of the gastrointestinal microbiota of high-altitude Tibetan pigs and low-altitude black pigs and identifies microbial taxa and functions that warrant further investigation under controlled experimental conditions.

## 2. Materials and Methods

### 2.1. Animal Management and Sample Collection

The Tibetan pigs and black pigs were raised separately in their respective native environments. Six Tibetan pigs were raised in Daocheng, Sichuan Province, China (28°26′ N, 100°19′ E; elevation approximately 3750 m), whereas six native Sichuan black pigs were raised in Suining, Sichuan Province, China (30°53′ N, 105°59′ E; elevation approximately 500 m). Following a 300-day stabilization period, all the pigs were transported to a nearby commercial slaughterhouse. All pigs were housed individually, with one pig per pen. Each pen had a floor area of approximately 3 m^2^. The pigs had free access to clean water and drinking devices that were cleaned daily. The growth data and feed formulations for the experimental pigs are detailed in Table S1. The pigs did not have diarrhea in the month preceding sampling, and no antibiotics were administered throughout the study. As per standard animal sampling protocols, the pigs underwent water deprivation for 2 h and fasting for 24 h prior to slaughter. We performed detailed dissections of each segment of their gastrointestinal tracts and collected samples of the contents of the stomach, jejunum, cecum, colon, and rectum. Immediately after collection, the samples were rapidly frozen in liquid nitrogen, kept on dry ice during transport to the laboratory, and then stored at −80 °C. A total of 60 gastrointestinal content samples were initially collected from the five gastrointestinal sites of six pigs per group, but three samples were excluded because they failed RNA quality control or sequencing quality criteria. Therefore, 57 samples were retained for downstream metatranscriptomic analyses (Table S2). All the procedures were conducted in accordance with the national guidelines for animal research institutions, and every effort was made to minimize distress. The study was approved by the Institutional Animal Care and Use Committee of Sichuan Animal Science Academy (Approval No. 2024024).

### 2.2. RNA Extraction and Quality Assessment

RNA was extracted from approximately 200 μL of each sample using QIAzol Lysis Reagent (Qiagen, Hilden, Germany), according to the manufacturer’s protocol, with minor modifications to improve microbial RNA recovery [20]. The samples were homogenized, incubated with chloroform, and then centrifuged to separate the aqueous phase. Genomic DNA was removed using a DNA elimination solution. RNA was then captured on silica spin columns, washed sequentially with RWT and RPE buffers, and eluted in RNase-free water. The RNA concentration of each sample was quantified using a Qubit RNA HS Assay Kit (Thermo Fisher Scientific, Waltham, MA, USA), and the integrity of the RNA was verified using an Agilent 2100 Bioanalyzer with the RNA 6000 Nano Kit (Agilent Technologies, Santa Clara, CA, USA) (RIN > 7). RNA purity was assessed using the A260/A280 ratio, aiming for ~2.0.

### 2.3. Library Construction and High-Throughput Sequencing

Ribosomal RNA was depleted using a Ribo-Zero rRNA Removal Kit (Illumina, San Diego, CA, USA) to enrich the samples for mRNA. First-strand cDNA synthesis was performed using an Ovation^®^ SoLo RNA-Seq Systems kit (Ovation^®^ SoLo RNA-Seq Systems; NuGEN Technologies, Inc., San Carlos, CA, USA), then second-strand synthesis was performed. The cDNA obtained was fragmented, ligated using sequencing adapters containing unique barcodes, and purified. Library quality was assessed using qPCR. High-throughput paired-end sequencing (PE150) was performed on the Illumina NovaSeq platform (Illumina, San Diego, CA, USA) by BGI (Shenzhen, China).

### 2.4. Data Preprocessing and Functional Annotation

FastQC was used to assess the raw data quality and to evaluate base quality distribution, GC content, and adapter contamination. Trimmomatic (v.0.39) was employed to remove low-quality bases (Q < 20) and adapter sequences. To minimize host DNA contamination, the quality-filtered reads were aligned against the *Sus scrofa* reference genome using Bowtie2 v.2.3.5, and reads that mapped to the host genome were removed. Residual rRNA sequences were removed after alignment against the SILVA database. The remaining high-quality microbial reads were assembled using Trinity, and redundant transcripts were clustered using Corset to generate nonredundant unigenes. The clean reads were then mapped back to the assembled reference to estimate gene abundance for downstream taxonomic and functional analyses. Functional annotation was performed using DIAMOND (v.0.8.35, e-values ≤ 1 × 10^−5^) and the Kyoto Encyclopedia of Genes and Genomes (KEGG) database, and HUMAnN3 was employed to identify the associated metabolic pathways.

### 2.5. Taxonomic Annotation of Metatranscriptomic Unigenes

Taxonomic annotation was performed using the assembled metatranscriptomic unigenes, which were aligned against the Micro_NR database using DIAMOND (v.0.8.35, e-values ≤ 1 × 10^−5^). The Micro_NR database was constructed from bacterial, fungal, archaeal, and viral sequences extracted from the NCBI NR database. Taxonomic assignments were determined using the lowest common ancestor algorithm, based on retained hits with e-values ≤ 10 times the minimum e-value. Taxonomic abundance profiles were generated at the kingdom, phylum, class, order, family, genus, and species levels and used for downstream microbial composition and differential abundance analyses.

### 2.6. Functional Annotation of CAZyme-Encoding Genes

Carbohydrate-active enzyme (CAZyme) annotation was performed by aligning the identified microbial gene sequences against the CAZy database using DIAMOND, with an e-value threshold of ≤1 × 10^−5^. The abundance of each CAZyme family was calculated by adding the normalized abundances of the genes assigned to that family. Annotated CAZyme families were further placed into the following major CAZyme classes: auxiliary activity (AA), carbohydrate-binding module (CBM), carbohydrate esterase (CE), glycoside hydrolase (GH), glycosyl transferase (GT), or polysaccharide lyase (PL).

### 2.7. Analysis of Microbial Diversity and Differential Bacterial Communities

Alpha diversity was quantified using the ACE, Chao1, Shannon, and Simpson indices. Differences in these indices between Tibetan pigs and Sichuan black pigs were evaluated separately within each gastrointestinal site using the Wilcoxon rank-sum test in R software (v4.3.2). Beta diversity was evaluated based on Bray–Curtis dissimilarities, and principal coordinates analysis (PCoA) and PERMANOVA were performed using the vegan package in R (v4.3.2) to provide an overall exploratory assessment of differences in the metatranscriptome-derived profiles among gastrointestinal sites. LEfSe analysis was conducted separately within each gastrointestinal site to identify differentially represented bacterial genera and species between Tibetan pigs and Sichuan black pigs. In each site-specific Wilcoxon rank-sum test and LEfSe analysis, each pig contributed only one observation and was considered the independent experimental unit. Taxa with *p* < 0.05 and an LDA score > 2.0 were considered differentially represented between the two groups.

### 2.8. Microbial Correlation Network Analysis

To identify associations among bacterial genera in the gastrointestinal microbiota, separate microbial correlation networks were constructed for the TPs and BPs using the genus-level relative abundance profiles obtained from the metatranscriptomic taxonomic annotation. Pairwise correlations among bacterial genera were calculated using Spearman’s rank correlation analysis in R software. Edge and node files were then generated in R software and imported into Gephi^®^ software (version 0.9.5) [21] for network visualization and topological analysis. In these networks, the nodes represent bacterial genera, and the edges represent significant genus–genus correlations. Network topological properties, including the number of nodes and edges, were calculated to compare the structural complexity of the gastrointestinal microbiota of the two pig groups.

## 3. Results

### 3.1. Overview of the Metatranscriptomes

To compare the gastrointestinal microbial profiles of Tibetan pigs and Sichuan black pigs, we compared the expression profiles of the gastrointestinal microbiota of TPs (high altitude, 3750 m, n = 6) and BPs (low altitude, 500 m, n = 6) at five anatomical sites (the stomach, jejunum, cecum, colon, and rectum). The metatranscriptomes (n = 57) of these samples were sequenced (Table S2). After removing the host-related sequences and filtering the reads for quality and length, a total of ~652.55 Gb of high-quality data were obtained, which represents approximately 11.45 Gb per sample on average. The base quality of the sequencing reads was high (Q30; base error < 0.1%; median Q30 = 92.44%) (Table S2). After the assembly of metatranscriptomic datasets, we identified a total of 6,838,229 transcripts and 3,010,464 non-redundant genes (unigenes).

### 3.2. Gastrointestinal Microbial Features of Tibetan Pigs and Black Pigs Revealed by Metatranscriptomic Analysis

**Figure 1:**
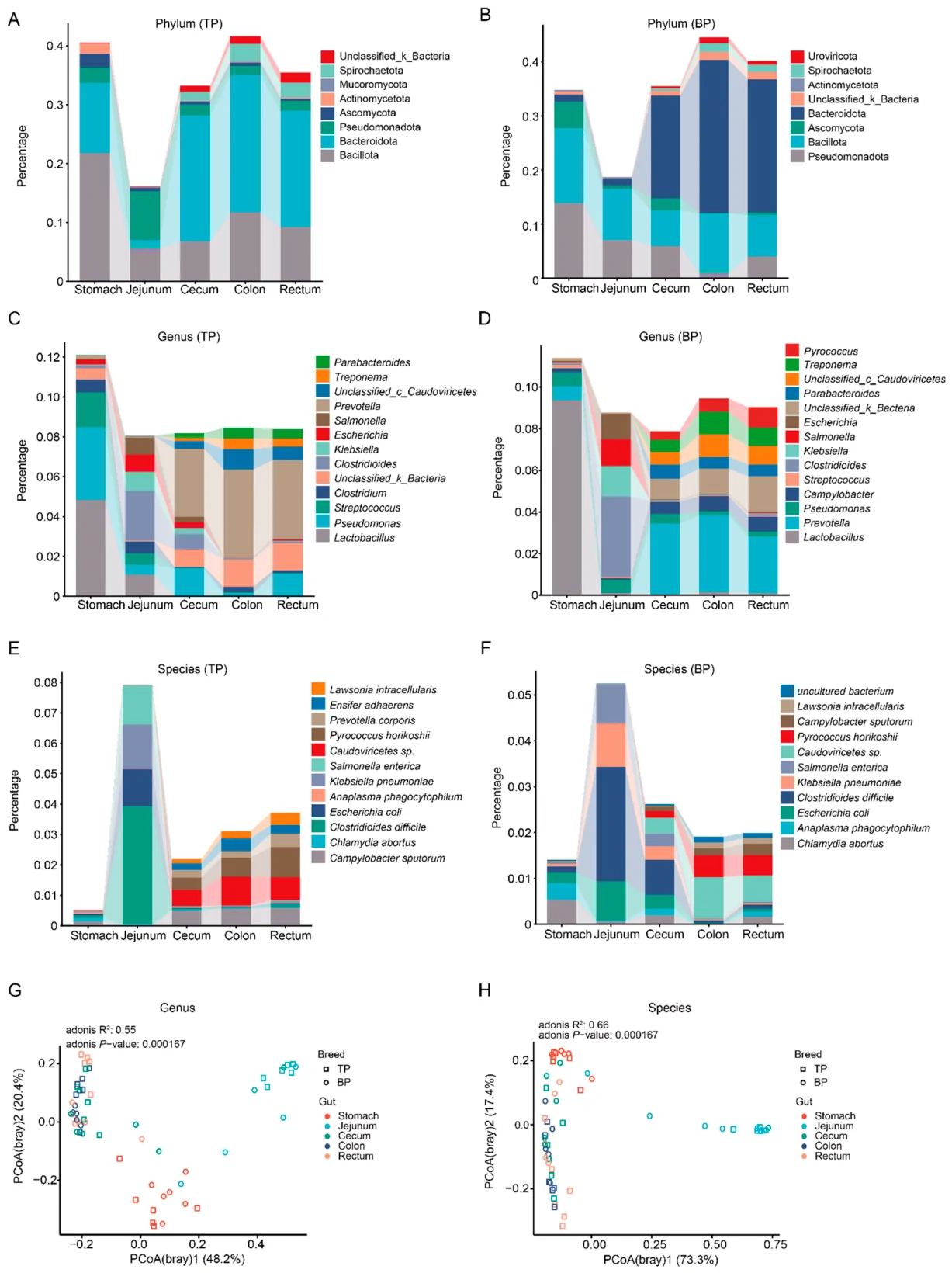
Composition of the gastrointestinal microbiota in Tibetan and black pigs: (**A**–**F**) Relative abundances of the top five most abundant microorganisms in the gastrointestinal segments of Tibetan pigs (TPs) and black pigs (BPs) at the phylum, genus, and species levels. (**G**,**H**) PCoA of samples, based on the Bray–Curtis distance, at the genus and species levels.

The metatranscriptomic sequencing data for bacteria, archaea, fungi, and viruses identified a total of 118 phyla, 1810 genera, and 5448 species. In Tibetan pigs, we found that Bacillota and Bacteroidota predominated in the stomach, whereas Pseudomonadota accounted for a relatively high proportion in the jejunum. The large intestine (cecum, colon, and rectum) was dominated by Bacteroidota and Bacillota (Figure 1A). At the genus level, *Lactobacillus* and *Pseudomonas* predominated in the stomach. *Clostridioides*, *Escherichia*, *Klebsiella*, and *Salmonella* were the predominant genera in the jejunum. In addition, *Prevotella* predominated throughout the large intestine (Figure 1C). The genus-level top 50 heatmap further showed that *Roseburia*, *Ruminococcus*, *Coprococcus*, and *Eubacterium* were more abundant in the large intestine than in the stomach and jejunum (Figure S1A). These anaerobic genera are commonly associated with dietary fiber fermentation and short-chain fatty acid production, suggesting a potential microbial contribution to the utilization of high-fiber diets in Tibetan pigs. At the species level, the predominant species in the jejunum included *Clostridioides difficile*, *Escherichia coli*, *Klebsiella pneumoniae*, and *Salmonella enterica* (Figure 1E). Interestingly, we found that *Caudoviricetes* sp. (belonging to bacteriophages) were the predominant species in the three parts of the large intestine in both pig groups, suggesting that bacteriophages may play an important role in the large intestine of pigs. In addition, *Eubacterium ventriosum*, *Blautia obeum*, *Coprococcus comes*, and *Anaerostipes caccae* had higher relative abundances in the large intestine than in the stomach and jejunum (Figure S1B). These species are associated with acetate and butyrate production that may benefit energy metabolism in intestinal epithelial cells and intestinal barrier homeostasis.

In black pigs, Bacillota and Pseudomonadota were the predominant phyla in the stomach and jejunum, whereas Bacteroidota predominated in the large intestine (Figure 1B). At the genus level, *Lactobacillus* was the predominant genus in the stomach. In the jejunum, *Clostridioides* was the most abundant genus, followed by *Klebsiella*, *Escherichia*, and *Salmonella*. *Prevotella* was the predominant genus throughout the large intestine (Figure 1D). *Chlamydia abortus* was the most abundant species in the BP stomach, whereas *Clostridioides difficile* was the most abundant species in the jejunum (Figure 1F). The genus- and species-level top 50 heatmaps further supported the marked variation in dominant taxa among gastrointestinal sites, broadly resembling the pattern observed in Tibetan pigs (Figure 1A,B). This similarity indicates that the spatial organization of the gastrointestinal tract is a major determinant of microbial community structure and that site-specific microbial profiles may reflect the distinct physicochemical conditions and physiological functions of different intestinal compartments.

Next, we compared the microbial structure of the samples by assessing the α- and β-diversity, based on the relative abundances of all the identified taxa at the genus and species levels. Beta-diversity, assessed using PCoA (the Bray–Curtis method), revealed that the samples primarily clustered according to the gastrointestinal site (Figure 1G,H): there was a clear separation of the stomach, jejunum, and large intestine (*p* < 0.00024), but no significant differences among the cecum, colon, and rectum (*p* > 0.09). The differences among the gastrointestinal sites were marked, but there were also differences in a few taxa between TPs and BPs, particularly between the stomachs of the pigs in these groups.

### 3.3. Microbial Diversity in the Gastrointestinal Tracts of the TPs and BPs

**Figure 2:**
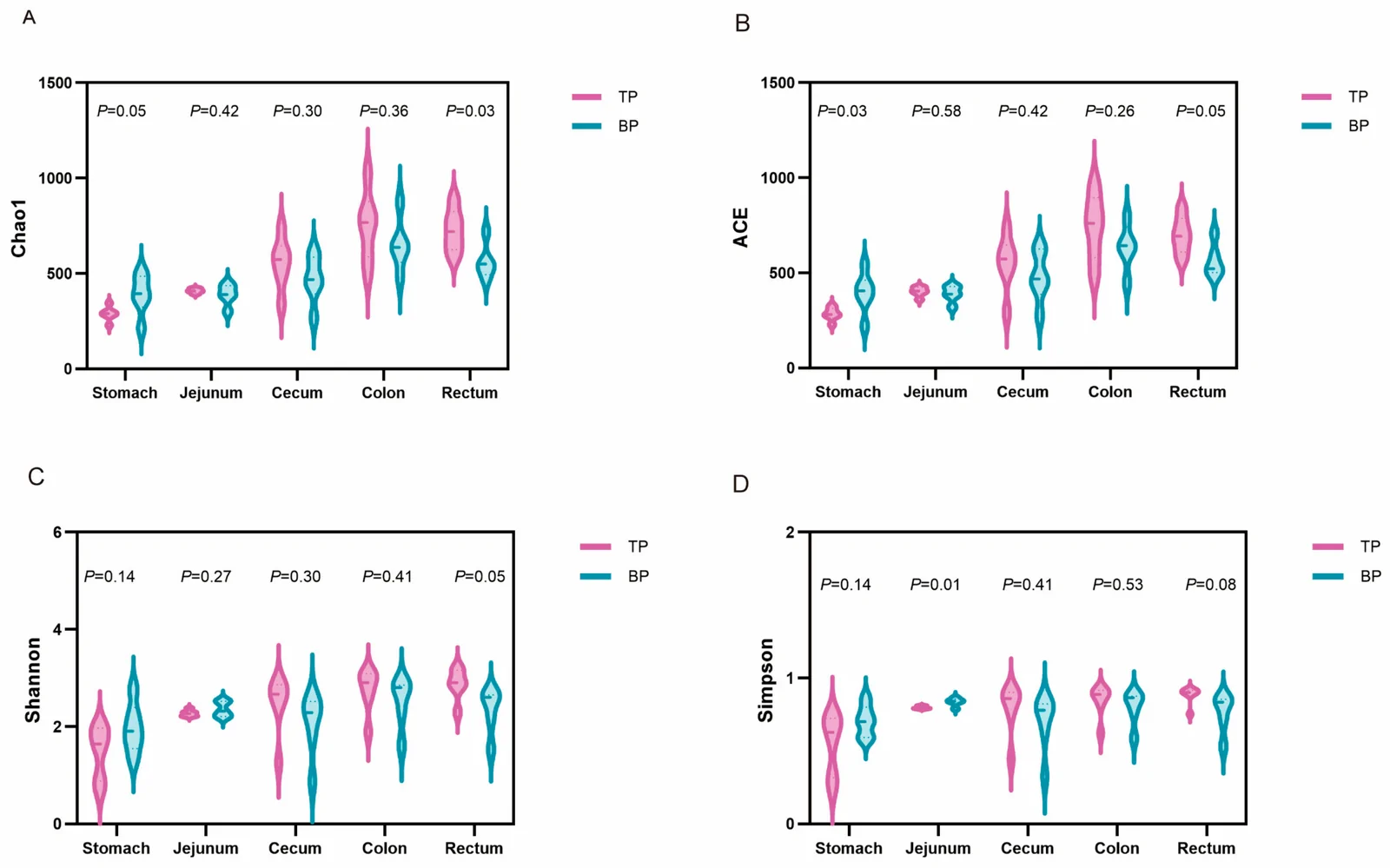
Alpha-diversity indices of the gut microbial genera in Tibetan and Black pigs: (**A**) Chao 1 index. (**B**) ACE index. (**C**) Shannon index. (**D**) Simpson index.

The α-diversity indices of the gastrointestinal microbiota in TPs and BPs obtained from the sequencing analysis data are shown in Figure 2. For the stomach content samples, the ACE index of the BP group was significantly higher than that of the TP group (*p* < 0.05), whereas in the rectum, the ACE index for the TP group was significantly higher than that for the BP group (*p* = 0.05). The Chao1 richness in the stomach of the BP group was higher than that of the TP group (*p* = 0.05), whereas the rectal Chao1 value was higher for the TP group (*p* < 0.05). The Simpson diversity in the jejunum was significantly higher for the BP group than for the TP group (*p* < 0.05). Overall, these results indicate gastrointestinal segment-specific differences in microbial α-diversity between the TPs and BPs.

### 3.4. Differences in Microbial Pathways Between TPs and BPs

**Figure 3:**
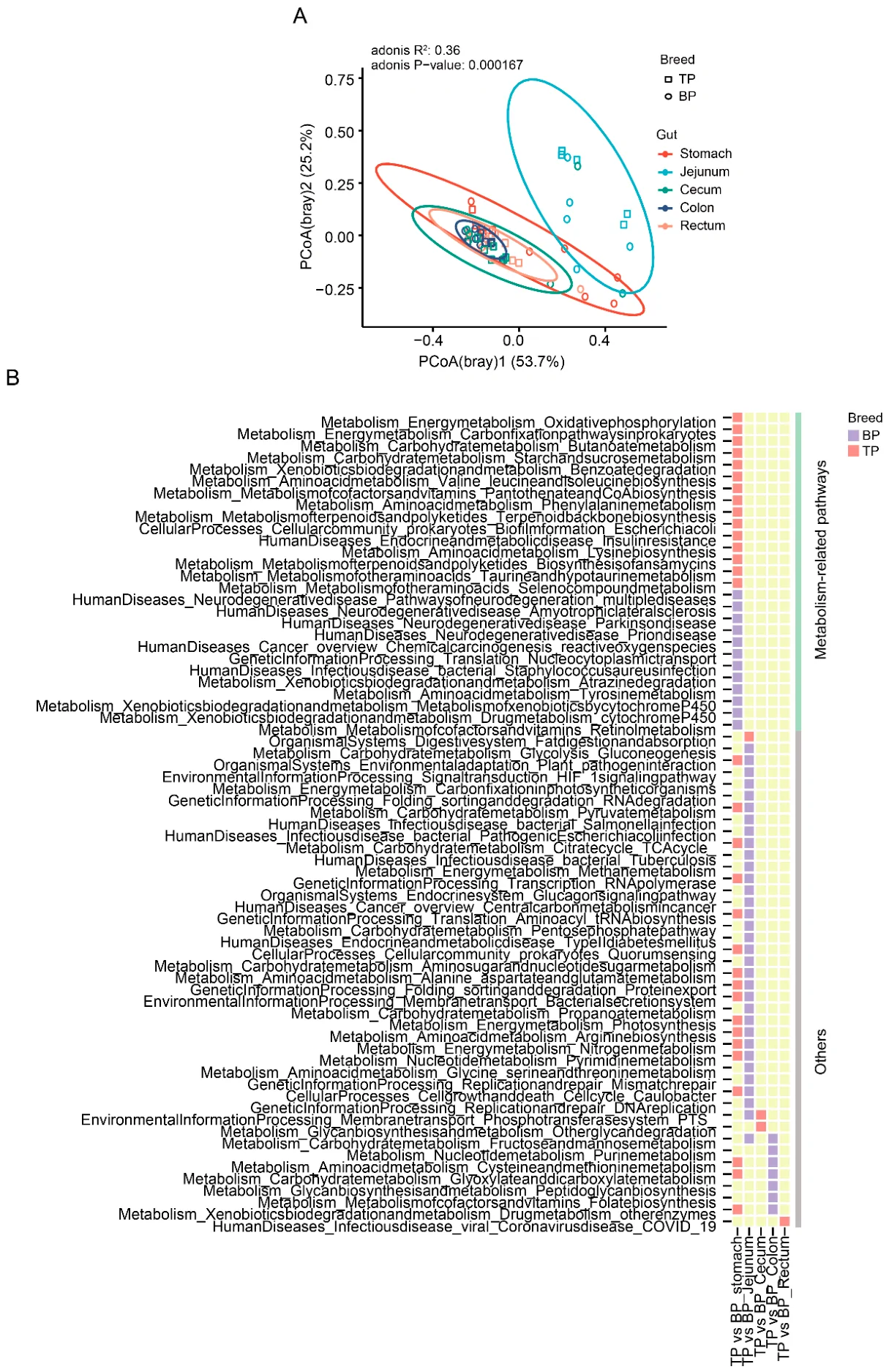
Functional differences in the gastrointestinal microbiota of Tibetan and black pigs: (**A**) PCoA diagram of the functional genes in the gastrointestinal microbiota of Tibetan and black pigs, based on the Bray–Curtis distance. (**B**) Heat map of the differential functions of the GI microbiota, based on KEGG pathway enrichment and LEfSe analysis, in Tibetan and black pigs.

In the gastrointestinal metatranscriptomes, 458 KEGG level-3 pathways were identified, and these microbial pathways were assigned to 46 level-2 categories. The overall functional PCoA based on the gene expression levels implied functional differences among the different parts of the gastrointestinal tract (Figure 3A). We next compared the abundances of KEGG pathways for BPs and TPs and for each gastrointestinal segment. Sixty-nine differentially abundant pathways (*p* < 0.05) were identified for BPs and TPs. These pathways were mainly associated with metabolism, including energy metabolism, carbohydrate metabolism, amino acid metabolism, nucleotide metabolism, xenobiotic biodegradation and metabolism, and glycan biosynthesis and metabolism (Figure 3B).

We then focused on the 40 differentially expressed metabolism-related pathways, ~69% of which were identified through the pairwise comparison for the stomach (Figure 3B). Of these, four energy metabolism pathways, five carbohydrate metabolism pathways, eight amino acid metabolism pathways, and one nucleotide metabolism pathway were more abundant (*p* < 0.05) in the TP stomach than in the BP stomach, suggesting that multiple aspects of metabolism differ at high altitude. These included carbohydrate metabolism pathways involved in starch and sucrose metabolism, glyoxylate and dicarboxylate metabolism, and butanoate metabolism. Because the metabolism of glucose requires less oxygen than that of fatty acids (a component of fats) and amino acids (a component of proteins), the upregulation of these pathways might contribute to metabolic adaptation to the hypoxic environment at high altitude. Moreover, pathways involved in amino acid metabolism, including cysteine and methionine metabolism, arginine biosynthesis, and leucine and isoleucine biosynthesis, were also more abundant in the TP stomach than in the BP stomach. Although the majority of the upregulated metabolic pathways in TPs were found in the stomach, a few metabolic pathways were also upregulated in the intestine of the TPs, including fat digestion and absorption and glycan degradation.

### 3.5. Differences in the Microbial Community Composition Between TPs and BPs

**Figure 4:**
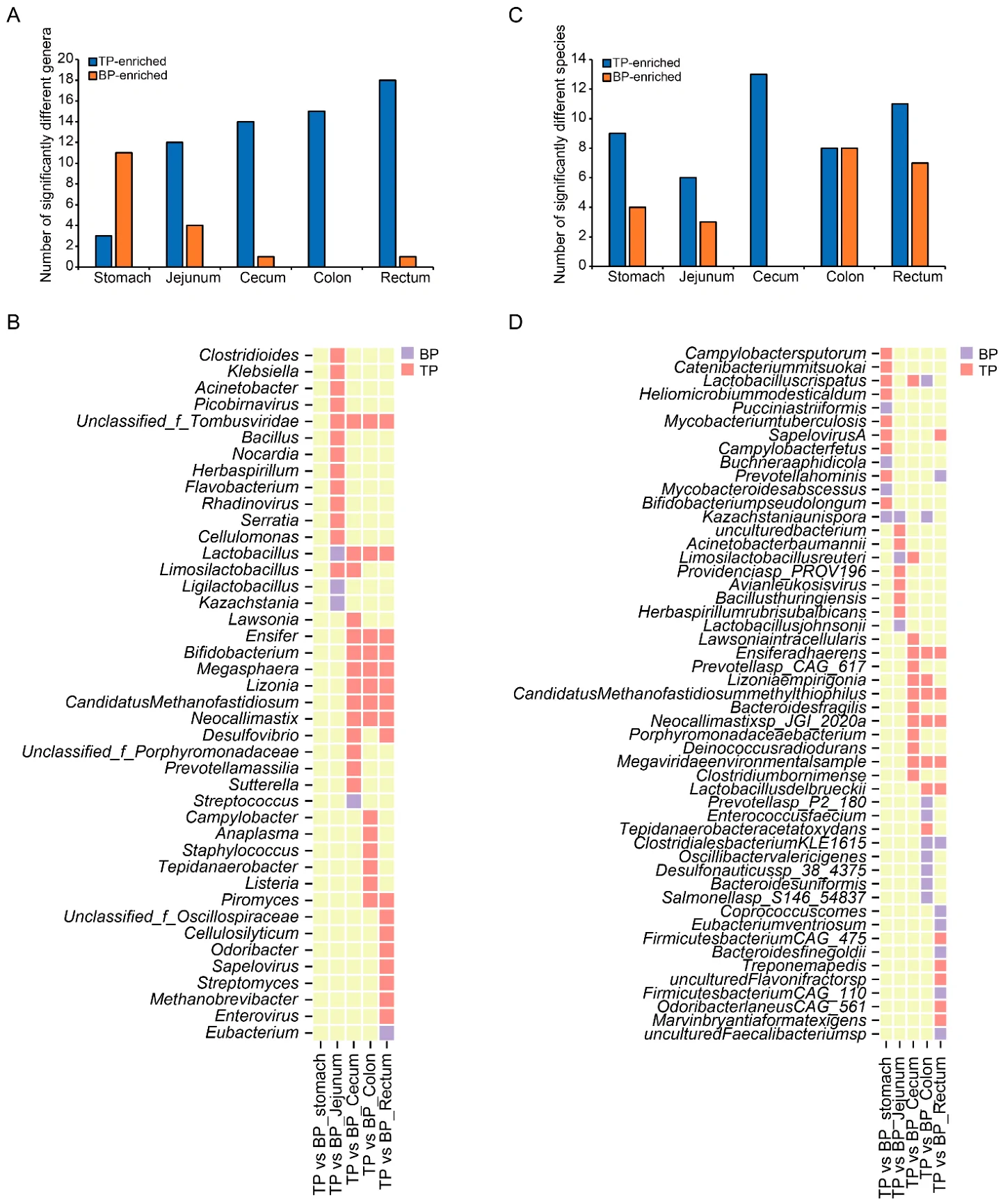
Taxa with the largest differences in abundance between Tibetan and black pigs: (**A**,**C**) Number of genera (**A**) and species (**C**) that significantly differed in abundance (*p* < 0.05, LDA > 2) between the Tibetan and black pigs in each of the five gastrointestinal segments. (**B**,**D**) Differentially abundant genera (**B**) and species (**D**), identified using LEfSe analysis, in Tibetan and black pigs (*p* < 0.05, LDA > 2).

We identified a total of 79 significantly differentially abundant genera between the TPs and BPs, ranging from 14 to 19 for each of the five gastrointestinal sites (Figure 4A). In the stomach, 14 genera differed in their relative abundance between the TPs and BPs, with three genera being more abundant in TPs and 11 being more abundant in BPs. Notably, of the 44 differentially abundant genera identified in the intestine (the jejunum, cecum, colon, and rectum), the vast majority (40 genera, ~91%) had higher relative abundances in TPs than in BPs (Figure 4B).

**Figure 5:**
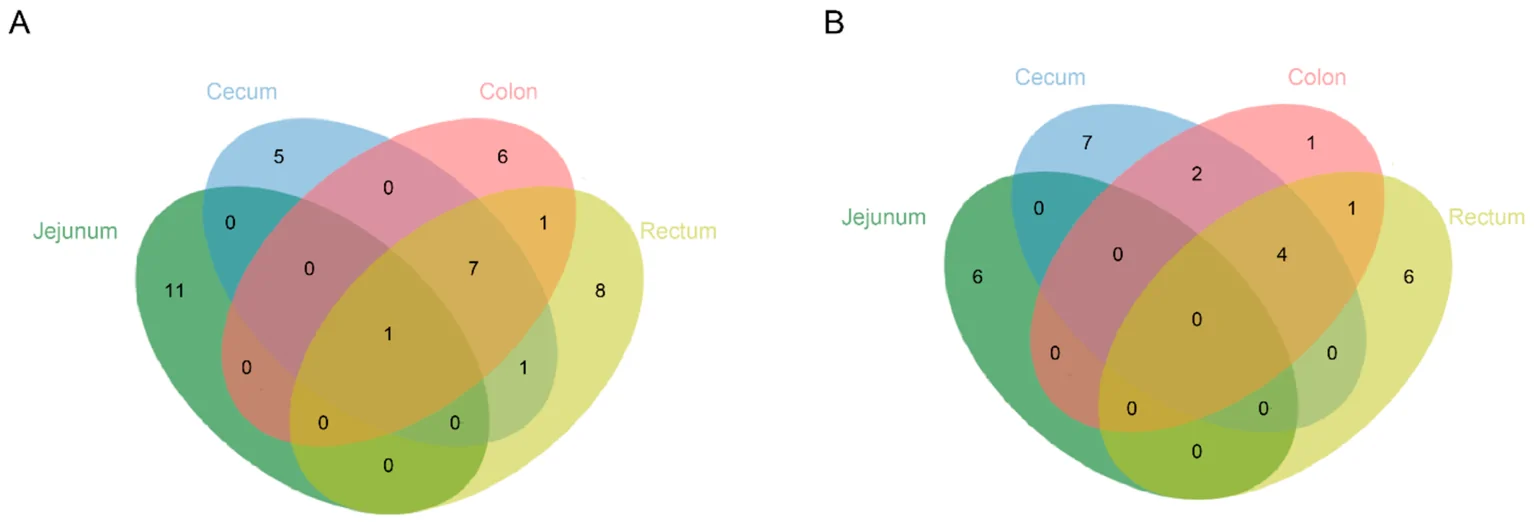
Venn diagram of the differentially abundant genera (**A**) and species (**B**) for the four intestinal sites.

Next, we assessed the distribution of the TP-enriched genera across the four intestinal segments. Most of these genera (30 of 40, 75%) showed segment-specific differences between the two pig populations (Figure 5A). Additionally, the genus *Unclassified_ f_ Tombusviridae* was enriched in both the jejunum and large intestine (cecum, colon, and rectum) of the TPs, but eight genera were enriched in all three large intestinal segments of the TPs, including three bacterial genera (*Lactobacillus*, *Bifidobacterium*, and *Megasphaera*). Interestingly, these genera are all involved in the synthesis of SCFAs, which could help the pigs with the energy efficiency necessary in high-altitude and harsh environments. Additionally, *Lactobacillus* and *Bifidobacterium* are the genera that are most commonly used as probiotics, which can improve intestinal barrier function and also play a role in enhancing immune competence. The greater relative abundances of these two bacterial genera in TPs may help to explain the previously identified stronger disease resistance in high-altitude TPs versus low-altitude pig populations.

Similar to the findings at the genus level, we found that in most gut segments, the TPs had more bacterial species with significantly higher relative abundances than the BPs, and this was especially true in the cecum (Figure 4C). Additionally, the number of TP-enriched species was comparable to the number of TP-enriched genera (40 genera vs. 34 species). Most of the identified TP-enriched species were segment-specific (Figure 4D and Figure 5B). We found that two species of the genus *Lactobacillus* were more abundant in the gastrointestinal tract of TPs: *Lactobacillus crispatus* was more abundant in the stomach and cecum of TPs than in BPs, whereas *Lactobacillus delbrueckii* was more abundant in the colon and rectum.

**Figure 6:**
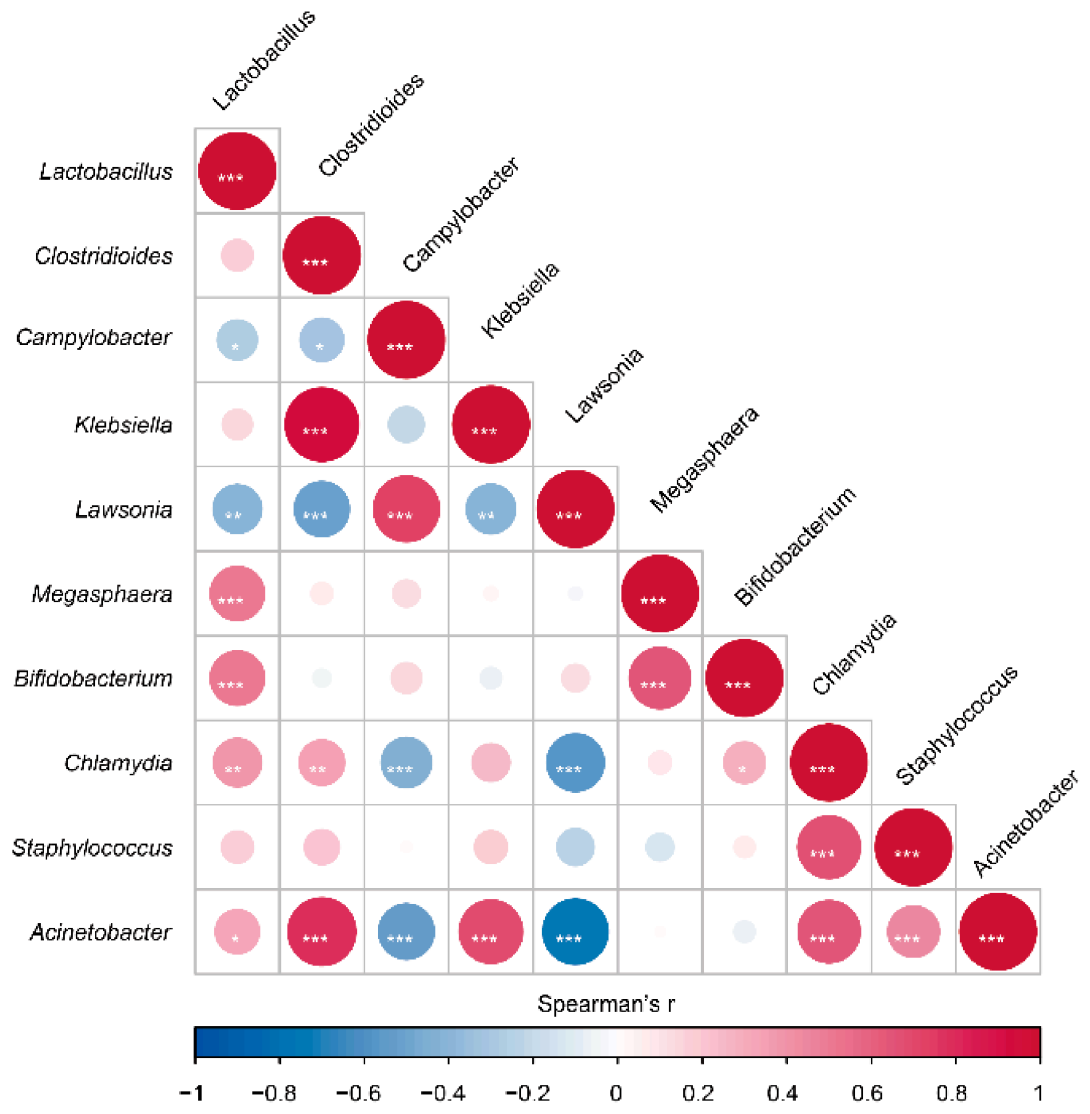
Heat map showing the Spearman’s rank correlations for the relationships among the 10 bacterial genera with the highest levels of enrichment in TPs vs. BPs (* *p* < 0.05, ** *p* < 0.01, *** *p* < 0.001).

**Figure 7:**
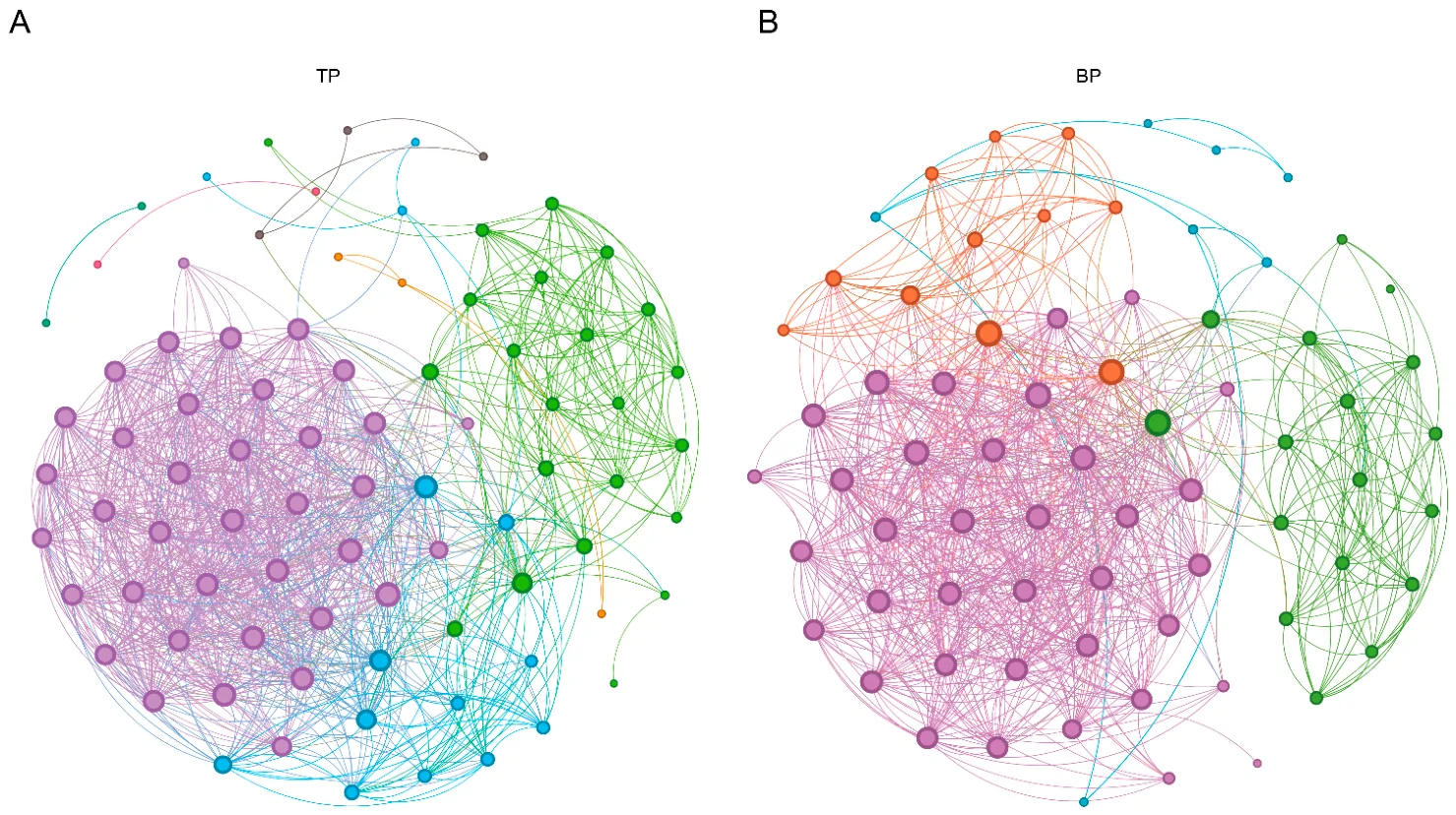
Correlation network diagrams for the GI microbiome in Tibetan pigs (**A**) and black pigs (**B**), based on the relative abundances of the bacterial genera.

We next characterized the relationships between the TP-enriched bacterial genera through correlation analysis. We found that the abundances of many of the TP-enriched genera positively and significantly correlated with each other, including *Lactobacillus* and *Megasphaera* (Figure 6). Next, we constructed genus-level bacterial correlation networks separately for TPs and BPs to further compare the potential microbial associations in the two pig groups. The results revealed that the TP gastrointestinal microbiome was slightly more complex than that of BPs (nodes: 83 vs. 71; edges: 1012 vs. 782) (Figure 7), suggesting that there are more complex microbial relationships in the gastrointestinal microbiota of TPs. However, whether this difference is associated with greater ecological stability or resilience requires further experimental validation.

### 3.6. Differences in the Expression of CAZyme-Encoding Genes

**Figure 8:**
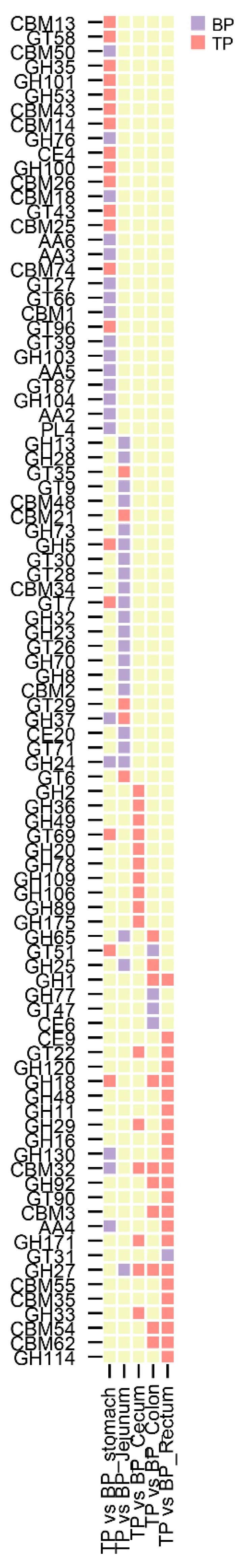
CAZyme-encoding genes showing differential expression between Tibetan and black pigs.

Four hundred seven CAZyme families were detected (17 AAs, 89 CBMs, 19 CEs, 156 GHs, 96 GTs, and 30 PLs), and GH and GT were the two most abundant classes in the gastrointestinal tracts of TPs and BPs. Several differences in the expression of CAZyme family genes were found in the TPs vs. the BPs. There were significant differences in the expression of 93 CAZyme families in at least one pairwise comparison between TP and BP segments (Figure 8). Of these, 57 were more abundant in the segments of TPs (1 AA, 13 CBMs, 2 CEs, 30 GHs, and 11 GTs), with the GH class showing the largest number of differences between TPs and BPs. We found that the differentially expressed CAZyme families were often more abundant in the TP than in the BP large intestine (cecum: 16 families upregulated in TPs vs. 0 in BPs, colon: 10 families upregulated in TPs vs. 4 in BPs, and rectum: 22 families upregulated in TPs vs. 1 in BPs), but this tendency was not replicated in the stomach (19 families upregulated in TPs vs. 20 in BPs) or jejunum (5 families upregulated in TPs vs. 22 in BPs). These findings suggest that the large intestinal microbiota of TPs is characterized by higher expression of CAZyme-encoding genes (Figure 8).

We next focused on the CAZyme family genes that were enriched in the large intestine. The specific differences were as follows: CE9, GH2, GH36, GH49, GT69, GH20, GH29, GH78, GH109, CBM32, GH171, GH27, GH106, GH33, GH89, and GH175 were upregulated in the TP cecum vs. the BP cecum; GH65, GH25, GH1, GH18, CBM32, GH92, CBM3, GH27, CBM54, and CBM62 were upregulated in the TP colon; and CE9, GT22, GH120, GH18, GH48, GH11, GH29, GH16, GH130, CBM32, GH92, GT90, CBM3, AA4, GH171, GH27, CBM55, CBM35, GH33, CBM54, CBM62, and GH114 were upregulated in the TP rectum. Among these differentially expressed CAZyme families, the transcripts assigned to CBM32 (binding to LacNAc (β-D-galactosyl-1,4-β-D-N-acetylglucosamine) and GH27 (one α-galactosidase module) were upregulated in all three parts of the large intestine of the TPs.

## 4. Discussion

To investigate the role of the gut microbiota in the adaptation of TPs to high-altitude environments, we performed metatranscriptome sequencing on samples of the gastrointestinal contents from TPs at a high altitude and BPs at a low altitude. We found that the abundances of the Firmicutes and Bacteroidota phyla in the stomach microbiota of the TPs were significantly higher than those of the BPs. Previous studies have shown that germ-free mice colonized with Firmicutes exhibit significantly lower body fat content, suggesting that this phylum may play a role in fat deposition in the host [22]. In addition, the Bacteroidota phylum is particularly effective at breaking down carbohydrates, which is consistent with the TPs’ ability to tolerate coarse feed [23,24].

We found that *Lactobacillus* and *Prevotella* were significantly enriched in the intestines of the TPs, a finding that is consistent with the results of a previous metagenomic study of the gastrointestinal microbiota of TPs [25]. Interestingly, in contrast with the metagenomic data obtained previously for high-altitude TPs and low-altitude BPs [25], the present metatranscriptomic analysis identified the genera *Streptococcus* and *Parabacteroides* in the gastrointestinal tracts of TPs and BPs. These microbial genera are closely associated with energy metabolism and the regulation of intestinal homeostasis. Specifically, *Streptococcus* is typically associated with the rapid fermentation of carbohydrates and lactic acid production; the lactic acid it produces can be used or converted to SCFAs, thereby contributing to energy metabolism in the gut [26]. *Parabacteroides* belongs to the Bacteroidetes phylum and potently degrades complex carbohydrates; it may participate in host energy metabolism and the regulation of intestinal homeostasis through polysaccharide use, succinate production, and bile acid metabolism [27].

Through our study of microbial interaction networks, we found that the gut microbiome of TPs is more complex (number of nodes: 83 vs. 71; number of edges: 1012 vs. 782), which may be involved in their adaptation to the stresses associated with high-altitude environments. Previous studies have shown that when a host is exposed to extreme conditions such as hypoxia, high pressure, or low temperature, its gut microbiota not only undergoes compositional changes but may also improve the adaptability of the host by increasing the stability of the microbial community and the complexity of the network [28,29]. Although greater microbial network complexity has been proposed to be associated with ecological stability in some studies, the present analysis did not directly assess temporal stability, resilience to environmental disturbance, or resistance to pathogen invasion. Therefore, the ecological implications of the observed network differences remain to be experimentally validated. Furthermore, we identified two beneficial bacterial species in TPs that help improve the host’s gut health (*Lactobacillus crispatus* [30]) and enhance nutrient absorption (*Lactobacillus delbrueckii* [31]). These findings may at least in part explain the high disease resistance of TPs.

An increase in the abundance of beneficial gut bacteria is typically associated with changes in metabolic processes. We identified a significant enrichment of various metabolic pathways in the stomach of the TPs, including energy metabolism, carbohydrate metabolism, and amino acid metabolism (including cysteine and methionine metabolism, arginine biosynthesis, and leucine and isoleucine biosynthesis), which is consistent with mammals requiring more energy at high altitude [32,33]. The enrichment of pathways related to amino acid metabolism in the stomach of TPs suggests that their gastric microbiota may be able to rapidly synthesize and transform amino acids. This may help TPs improve the efficiency with which they use nutrients and adapt their metabolism to high-altitude, hypoxic environments with relatively limited nutritional resources [34,35].

In addition to the differences in gastric metabolic pathways, we also found that the gut microbiota of TPs is significantly enriched in metabolic pathways related to nutrient utilization, such as fat digestion and absorption and glycan degradation. These findings suggest that the adaptation of microbial function by TPs is not restricted to changes in the stomach microenvironment but may contribute to high-altitude adaptation through coordinated changes in metabolism across multiple gastrointestinal segments. Consistent with these findings, the gene expression of CAZymes, which are involved in carbohydrate degradation, in the large intestine of TPs was significantly higher than that of low-altitude BPs, suggesting that their large intestinal microbiota may have a higher capacity to use complex carbon sources. This difference may be related to differences in the composition of the gut microbiota between the two groups [36,37]. In particular, the increase in CBM32-related gene expression may increase the ability of microorganisms to recognize specific sugar chains, such as LacNAc [38], and the increase in expression of GH27 family genes facilitates the hydrolysis of α-galactoside substrates and the release of monosaccharides that can be used by the gut microbiota of TPs [39]. This may be part of the adaptation of TPs to cope with the high-fiber, low-nutrient environment of the plateau [40].

High altitudes can cause specific adaptive changes in the microbial communities of the various gastrointestinal segments of the host [41]. In the present study, gastrointestinal microbial composition also differed between high-altitude Tibetan pigs and low-altitude black pigs. The microbial communities of the stomach and jejunum of the TPs had similar compositions and were dominated by the Firmicutes and Bacteroidetes, which are associated with nutrient digestion, carbohydrate fermentation, and microbial energy metabolism [42,43]. In addition, the abundance of anaerobic microorganisms in the gut microbiota of TPs, including in the cecum, colon, and rectum, is higher than that of BPs, such as *Lactobacillus*, *Bifidobacterium*, and *Megasphaera*. These microbial groups contribute to SCFA synthesis and the maintenance of the intestinal barrier, and their higher relative abundances in Tibetan pigs may be related to differences in nutrient utilization and the intestinal environment between the two groups [44,45]. However, these associations do not demonstrate that the observed microbial differences were caused by altitude or contributed directly to high-altitude adaptation. Taken together, the differences in the microbial composition of the segments of the gastrointestinal tract support the view that TPs may adapt to high-altitude environments by reshaping the microbial composition and metabolic functions of these segments [46,47]. Overall, these results suggest that the gut microbiota may be closely associated with the efficiency of host nutrient use and metabolic adaptation in high-altitude environments. Taken together, the gastrointestinal microbial communities differed between Tibetan pigs and black pigs. Future studies should compare different pig populations at the same altitude to better distinguish the effects of population from environmental factors.

## 5. Conclusions

In this study, we characterized differences in gastrointestinal microbial composition and transcriptional functions between high-altitude Tibetan pigs and low-altitude black pigs. *Lactobacillus crispatus* and *Lactobacillus delbrueckii* showed higher relative abundances in the gastrointestinal microbiota of Tibetan pigs. Microbial pathways related to energy, carbohydrate, amino acid, and nucleotide metabolism were also more abundant in Tibetan pigs, together with higher abundances of the CAZyme families CBM32 and GH27. These microbial and functional features were associated with high-altitude Tibetan pigs and may be related to nutrient utilization and energy metabolism under high-altitude conditions. However, because the present study was observational, these associations do not demonstrate that the identified microbial features causally contribute to high-altitude adaptation. Further controlled experiments and functional validation are required to determine their roles in this process.

## Data Availability

The gastrointestinal metatranscriptomic sequencing data generated in this study have been deposited in the NCBI Sequence Read Archive (SRA) database under BioProject accession number PRJNA1193123.

## Supplementary Materials

The following supporting information can be downloaded at https://www.mdpi.com/article/10.3390/ani16182861/s1, Figure S1: Heatmaps of the top 50 microbial taxa across gastrointestinal sites in Tibetan and black pigs; Table S1: Black pig and Tibetan pig growth data; Table S2: Sequencing data information for each individual; Table S3: Detailed information on the 57 gastrointestinal content samples used for metatranscriptomic sequencing and their corresponding NCBI BioSample and SRA Run accession numbers.
