# Supplementary material for: Metatranscriptomic Analysis Reveals Functional Features of the Gastrointestinal Microbiota in Tibetan Pigs
Source: Animals (Basel). 2026 Sep 11;16(18):2861. doi: 10.3390/ani16182861 (PMC13603674; doi:10.3390/ani16182861)
Supplement: Supplementary file 1 [file animals-16-02861-s001.zip › Supplementary Figure S1.pdf]

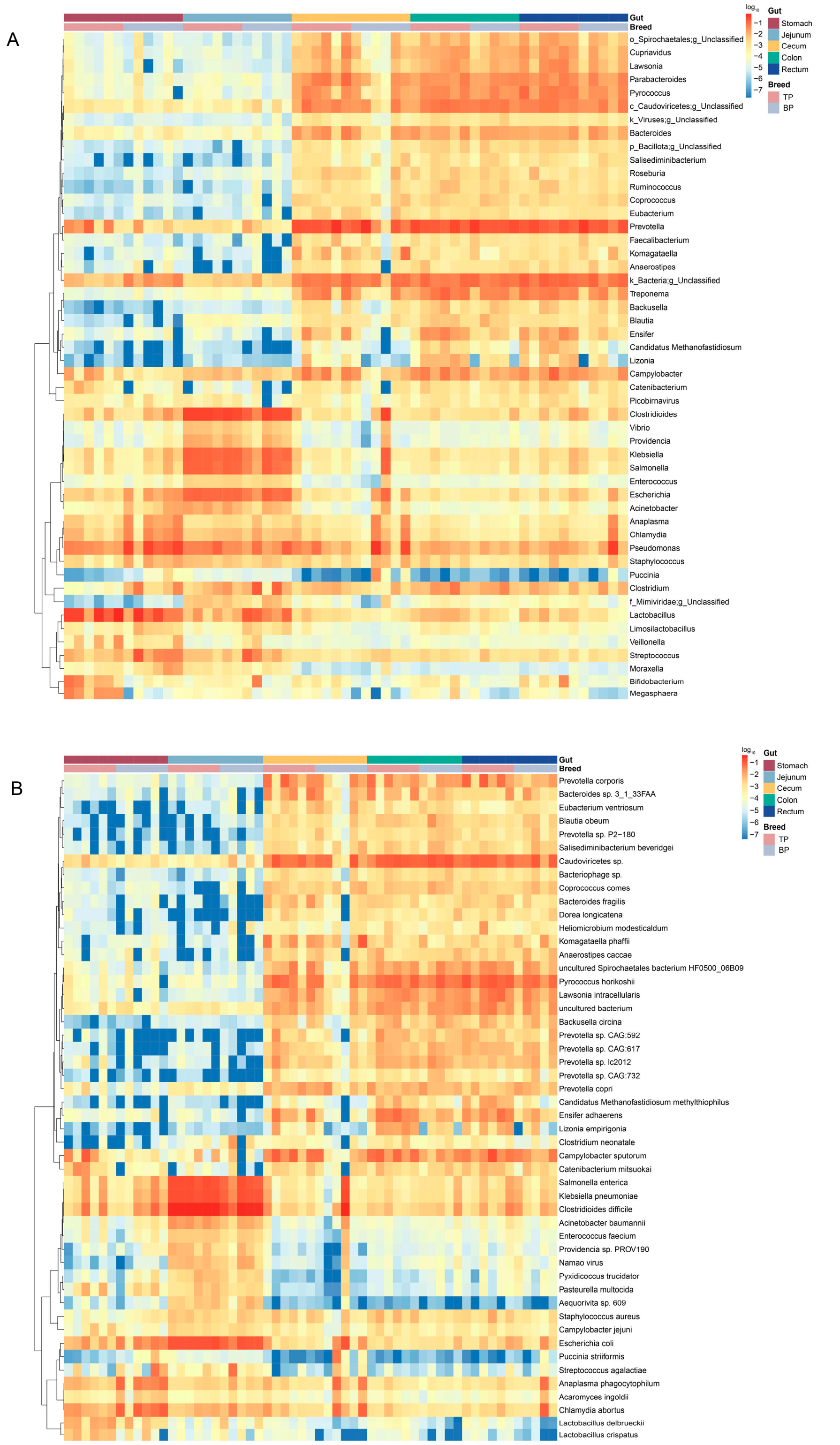

Figure S1. Heatmaps of the top 50 microbial taxa across gastrointestinal sites in Tibetan and black pigs. (A) Genus-level heatmap; (B) species-level heatmap.
