# Supplementary material for: Metatranscriptomic Analysis Reveals Functional Features of the Gastrointestinal Microbiota in Tibetan Pigs
Source: Animals (Basel). 2026 Sep 11;16(18):2861. doi: 10.3390/ani16182861 (PMC13603674; doi:10.3390/ani16182861)
Supplement: Supplementary file 1 [file animals-16-02861-s001.zip › Table S1.pdf]

Table S1 Black pig and Tibetan pig growth data					
**SampleID**	**Sample original name**	**Breed**	**Weight**	**Back fat**	**Daily weight gain**
TP1	LQ1	TP	52.7	18	228.97
TP2	LQ2	TP	50.2	16	229.76
TP3	LQ3	TP	51.5	17	229.73
TP4	LQ4	TP	45.3	19	231.36
TP5	LQ5	TP	46.4	16	226.49
TP6	LQ6	TP	48.8	17	225.38
BP1	SN1	BP	89.3	13.8	565.63
BP2	SN2	BP	89.2	16.5	588.77
BP3	SN3	BP	93.3	16.6	612.08
BP4	SN4	BP	90.1	16.2	549.1
BP5	SN5	BP	91	15.9	601.57
BP6	SN6	BP	89.2	13	576.52

Diet composition

Feeding a uniformly formulated basic diet, the diet composition is as follows:

Energy feed: corn accounts for about 60%;

Protein feed: soybean meal accounts for about 25%;

Crude fiber feed: bran and silage account for about 10%;

Mineral and vitamin additives: account for about 5%.

Feeding environment

Each pig was provided with a feeding space of 3.0 m^2/head to ensure sufficient room for activities.

Temperature control: Black pigs were raised at low altitudes (500 m above sea level), with a naturally changing ambient temperature ranging from 15-25°C, and equipped with sunshade and ventilation equipment to cope with hot weather; Tibetan pigs were raised at high altitudes (3750 m above sea level), with an ambient temperature of 5-15°C, and provided with insulation facilities to ensure the comfort of pigs in cold weather. According to long-term local climatological records, the mean outdoor relative humidity during the corresponding sampling period was approximately 62% and 84%

Bedding: Straw was provided as bedding during feeding and was changed regularly to keep the pens clean and dry.
