# Supplementary material for: Metatranscriptomic Analysis Reveals Functional Features of the Gastrointestinal Microbiota in Tibetan Pigs
Source: Animals (Basel). 2026 Sep 11;16(18):2861. doi: 10.3390/ani16182861 (PMC13603674; doi:10.3390/ani16182861)
Supplement: Supplementary file 1 [file animals-16-02861-s001.zip › Table S2.pdf]

Sample Name	Breed	Tissue	Clean Reads	Clean Base (Gb)	Read Length	Q20(%)	Q30(%)	GC(%)
LQ1_cecumco_3A	Tibetan pig	Cecum	34,025,737	10.21	PE150	97.15	91.21	55.73
LQ1_colonco_3A	Tibetan pig	Colon	40,155,676	12.05	PE150	97.83	92.49	49.65
LQ1_jejuco_3A	Tibetan pig	Jejunum	40,148,948	12.04	PE150	96.92	89.76	46.75
LQ1_rectco_3A	Tibetan pig	Rectum	37,385,048	11.22	PE150	98.02	93.11	48.90
LQ1_stomco_3A	Tibetan pig	Stomach	37,813,550	11.34	PE150	97.59	92.02	56.69
LQ2_cecumco_3A	Tibetan pig	Cecum	35,666,210	10.70	PE150	97.73	92.00	47.81
LQ2_colonco_3A	Tibetan pig	Colon	40,056,866	12.02	PE150	97.62	91.71	48.17
LQ2_jejuco_3A	Tibetan pig	Jejunum	40,230,704	12.07	PE150	97.02	90.16	47.91
LQ2_rectco_3A	Tibetan pig	Rectum	40,131,935	12.04	PE150	97.61	91.50	47.02
LQ2_stomco_3A	Tibetan pig	Stomach	31,533,292	9.46	PE150	97.57	92.12	56.12
LQ3_cecumco_3A	Tibetan pig	Cecum	40,105,628	12.03	PE150	97.39	91.22	50.56
LQ3_colonco_3A	Tibetan pig	Colon	40,113,288	12.03	PE150	97.45	91.38	46.76
LQ3_jejuco_3A	Tibetan pig	Jejunum	40,167,598	12.05	PE150	97.11	90.44	47.57
LQ3_rectco_3A	Tibetan pig	Rectum	40,026,484	12.01	PE150	97.55	91.73	48.48
LQ3_stomco_3A	Tibetan pig	Stomach	38,089,565	11.43	PE150	97.40	91.57	55.88
LQ4_cecumco_3A	Tibetan pig	Cecum	40,146,333	12.04	PE150	97.52	91.48	47.92
LQ4_colonco_3A	Tibetan pig	Colon	40,045,816	12.01	PE150	97.41	90.97	45.56
LQ4_jejuco_3A	Tibetan pig	Jejunum	35,443,113	10.63	PE150	96.02	89.26	59.65
LQ4_rectco_3A	Tibetan pig	Rectum	40,079,771	12.02	PE150	97.51	91.45	48.22
LQ4_stomco_3A	Tibetan pig	Stomach	40,200,220	12.06	PE150	97.72	92.44	51.97
LQ5_cecumco_3A	Tibetan pig	Cecum	40,143,456	12.04	PE150	97.69	92.26	51.77
LQ5_colonco_3A	Tibetan pig	Colon	40,160,677	12.05	PE150	97.41	91.08	45.86
LQ5_jejuco_3A	Tibetan pig	Jejunum	34,533,188	10.36	PE150	96.79	89.73	48.81
LQ5_rectco_3A	Tibetan pig	Rectum	39,936,061	11.98	PE150	97.47	91.87	53.33

LQ5_stomco_3A	Tibetan pig	Stomach	40,118,578	12.04	PE150	97.38	91.39	54.08
LQ6_cecumco_3A	Tibetan pig	Cecum	40,114,581	12.03	PE150	97.45	91.38	48.62
LQ6_colonco_3A	Tibetan pig	Colon	40,084,892	12.03	PE150	98.28	93.70	47.13
LQ6_jejuco_3A	Tibetan pig	Jejunum	40,000,666	12.00	PE150	95.95	89.08	61.41
LQ6_rectco_3A	Tibetan pig	Rectum	40,179,000	12.05	PE150	98.28	93.83	47.75
LQ6_stomco_3A	Tibetan pig	Stomach	39,907,477	11.97	PE150	97.68	92.27	51.52
SN1_cecumco_3A	Scihuan black pig	Cecum	40,127,763	12.04	PE150	98.62	95.01	49.84
SN1_stomco_3A	Scihuan black pig	Stomach	30,472,587	9.14	PE150	97.77	92.22	53.48
SN2_cecumco_3A	Scihuan black pig	Cecum	40,094,357	12.03	PE150	98.53	94.61	49.10
SN2_colonco_3A	Scihuan black pig	Colon	40,022,915	12.01	PE150	98.46	94.40	49.69
SN2_jejuco_3A	Scihuan black pig	Jejunum	34,039,047	10.21	PE150	97.89	93.46	52.75
SN2_rectco_3A	Scihuan black pig	Rectum	40,216,200	12.06	PE150	98.36	94.33	51.62
SN2_stomco_2A	Scihuan black pig	Stomach	40,192,280	12.06	PE150	97.89	92.39	44.70
SN3_cecumco_3A	Scihuan black pig	Cecum	35,050,199	10.52	PE150	97.92	92.73	52.40
SN3_colonco_3A	Scihuan black pig	Colon	40,109,016	12.03	PE150	98.25	93.62	47.23
SN3_jejuco_3A	Scihuan black pig	Jejunum	32,829,025	9.85	PE150	97.15	92.47	57.51
SN3_rectco_3A	Scihuan black pig	Rectum	40,140,665	12.04	PE150	98.42	94.27	49.94
SN3_stomco_3A	Scihuan black pig	Stomach	35,736,260	10.72	PE150	97.40	92.44	56.13
SN4_cecumco_3A	Scihuan black pig	Cecum	39,486,836	11.85	PE150	97.84	92.72	48.28
SN4_colonco_3A	Scihuan black pig	Colon	40,092,494	12.03	PE150	98.47	94.39	47.90
SN4_jejuco_3A	Scihuan black pig	Jejunum	40,246,645	12.07	PE150	97.97	93.01	46.90
SN4_rectco_3A	Scihuan black pig	Rectum	40,172,942	12.05	PE150	98.52	94.53	46.79
SN4_stomco_3A	Scihuan black pig	Stomach	32,732,962	9.82	PE150	97.88	92.77	52.13
SN5_cecumco_3A	Scihuan black pig	Cecum	40,149,196	12.04	PE150	98.51	94.56	46.89
SN5_colonco_3A	Scihuan black pig	Colon	40,115,192	12.03	PE150	98.39	94.09	45.93
SN5_jejuco_3A	Scihuan black pig	Jejunum	40,081,083	12.02	PE150	97.99	93.63	50.29

SN5_rectco_3A	Scihuan black pig	Rectum	30,519,013	9.16	PE150	97.83	93.04	55.48
SN5_stomco_3A	Scihuan black pig	Stomach	30,285,165	9.09	PE150	97.81	93.78	54.69
SN6_cecumco_3A	Scihuan black pig	Cecum	35,374,192	10.61	PE150	98.13	94.11	55.72
SN6_colonco_3A	Scihuan black pig	Colon	40,073,770	12.02	PE150	98.43	94.36	48.62
SN6_jejuco_3A	Scihuan black pig	Jejunum	38,293,514	11.49	PE150	97.08	92.02	59.57
SN6_rectco_3A	Scihuan black pig	Rectum	40,154,539	12.05	PE150	98.34	93.99	48.17
SN6_stomco_3B	Scihuan black pig	Stomach	31,604,294	9.48	PE150	97.52	92.68	54.97

**Sample Name**	**Breed**	**Tissue**	**Clean Reads**	**Clean Base (Gb)**	**Read Length**	**Q20(%)**	**Q30(%)**	**GC(%)**
SN1_cecumco_3A	Scihuan black pig	Cecum	40,127,763	12.04	PE150	98.62	95.01	49.84
SN2_cecumco_3A	Scihuan black pig	Cecum	40,094,357	12.03	PE150	98.53	94.61	49.10
SN3_cecumco_3A	Scihuan black pig	Cecum	35,050,199	10.52	PE150	97.92	92.73	52.40
SN4_cecumco_3A	Scihuan black pig	Cecum	39,486,836	11.85	PE150	97.84	92.72	48.28
SN5_cecumco_3A	Scihuan black pig	Cecum	40,149,196	12.04	PE150	98.51	94.56	46.89
SN6_cecumco_3A	Scihuan black pig	Cecum	35,374,192	10.61	PE150	98.13	94.11	55.72
SN2_colonco_3A	Scihuan black pig	Colon	40,022,915	12.01	PE150	98.46	94.40	49.69
SN3_colonco_3A	Scihuan black pig	Colon	40,109,016	12.03	PE150	98.25	93.62	47.23
SN4_colonco_3A	Scihuan black pig	Colon	40,092,494	12.03	PE150	98.47	94.39	47.90
SN5_colonco_3A	Scihuan black pig	Colon	40,115,192	12.03	PE150	98.39	94.09	45.93
SN6_colonco_3A	Scihuan black pig	Colon	40,073,770	12.02	PE150	98.43	94.36	48.62

SN2_jejuco_3A	Scihuan black pig	Jejunum	34,039,047	10.21	PE150	97.89	93.46	52.75
SN3_jejuco_3A	Scihuan black pig	Jejunum	32,829,025	9.85	PE150	97.15	92.47	57.51
SN4_jejuco_3A	Scihuan black pig	Jejunum	40,246,645	12.07	PE150	97.97	93.01	46.90
SN5_jejuco_3A	Scihuan black pig	Jejunum	40,081,083	12.02	PE150	97.99	93.63	50.29
SN6_jejuco_3A	Scihuan black pig	Jejunum	38,293,514	11.49	PE150	97.08	92.02	59.57
SN2_rectco_3A	Scihuan black pig	Rectum	40,216,200	12.06	PE150	98.36	94.33	51.62
SN3_rectco_3A	Scihuan black pig	Rectum	40,140,665	12.04	PE150	98.42	94.27	49.94
SN4_rectco_3A	Scihuan black pig	Rectum	40,172,942	12.05	PE150	98.52	94.53	46.79
SN5_rectco_3A	Scihuan black pig	Rectum	30,519,013	9.16	PE150	97.83	93.04	55.48
SN6_rectco_3A	Scihuan black pig	Rectum	40,154,539	12.05	PE150	98.34	93.99	48.17
SN1_stomco_3A	Scihuan black pig	Stomach	30,472,587	9.14	PE150	97.77	92.22	53.48
SN2_stomco_2A	Scihuan black pig	Stomach	40,192,280	12.06	PE150	97.89	92.39	44.70
SN3_stomco_3A	Scihuan black pig	Stomach	35,736,260	10.72	PE150	97.40	92.44	56.13
SN4_stomco_3A	Scihuan black pig	Stomach	32,732,962	9.82	PE150	97.88	92.77	52.13
SN5_stomco_3A	Scihuan black pig	Stomach	30,285,165	9.09	PE150	97.81	93.78	54.69
SN6_stomco_3B	Scihuan black pig	Stomach	31,604,294	9.48	PE150	97.52	92.68	54.97
LQ1_cecumco_3A	Tibetan pig	Cecum	34,025,737	10.21	PE150	97.15	91.21	55.73
LQ2_cecumco_3A	Tibetan pig	Cecum	35,666,210	10.70	PE150	97.73	92.00	47.81
LQ3_cecumco_3A	Tibetan pig	Cecum	40,105,628	12.03	PE150	97.39	91.22	50.56
LQ4_cecumco_3A	Tibetan pig	Cecum	40,146,333	12.04	PE150	97.52	91.48	47.92
LQ5_cecumco_3A	Tibetan pig	Cecum	40,143,456	12.04	PE150	97.69	92.26	51.77
LQ6_cecumco_3A	Tibetan pig	Cecum	40,114,581	12.03	PE150	97.45	91.38	48.62
LQ1_colonco_3A	Tibetan pig	Colon	40,155,676	12.05	PE150	97.83	92.49	49.65
LQ2_colonco_3A	Tibetan pig	Colon	40,056,866	12.02	PE150	97.62	91.71	48.17
LQ3_colonco_3A	Tibetan pig	Colon	40,113,288	12.03	PE150	97.45	91.38	46.76
LQ4_colonco_3A	Tibetan pig	Colon	40,045,816	12.01	PE150	97.41	90.97	45.56

LQ5_colonco_3A	Tibetan pig	Colon	40,160,677	12.05	PE150	97.41	91.08	45.86
LQ6_colonco_3A	Tibetan pig	Colon	40,084,892	12.03	PE150	98.28	93.70	47.13
LQ1_jejuco_3A	Tibetan pig	Jejunum	40,148,948	12.04	PE150	96.92	89.76	46.75
LQ2_jejuco_3A	Tibetan pig	Jejunum	40,230,704	12.07	PE150	97.02	90.16	47.91
LQ3_jejuco_3A	Tibetan pig	Jejunum	40,167,598	12.05	PE150	97.11	90.44	47.57
LQ4_jejuco_3A	Tibetan pig	Jejunum	35,443,113	10.63	PE150	96.02	89.26	59.65
LQ5_jejuco_3A	Tibetan pig	Jejunum	34,533,188	10.36	PE150	96.79	89.73	48.81
LQ6_jejuco_3A	Tibetan pig	Jejunum	40,000,666	12.00	PE150	95.95	89.08	61.41
LQ1_rectco_3A	Tibetan pig	Rectum	37,385,048	11.22	PE150	98.02	93.11	48.90
LQ2_rectco_3A	Tibetan pig	Rectum	40,131,935	12.04	PE150	97.61	91.50	47.02
LQ3_rectco_3A	Tibetan pig	Rectum	40,026,484	12.01	PE150	97.55	91.73	48.48
LQ4_rectco_3A	Tibetan pig	Rectum	40,079,771	12.02	PE150	97.51	91.45	48.22
LQ5_rectco_3A	Tibetan pig	Rectum	39,936,061	11.98	PE150	97.47	91.87	53.33
LQ6_rectco_3A	Tibetan pig	Rectum	40,179,000	12.05	PE150	98.28	93.83	47.75
LQ1_stomco_3A	Tibetan pig	Stomach	37,813,550	11.34	PE150	97.59	92.02	56.69
LQ2_stomco_3A	Tibetan pig	Stomach	31,533,292	9.46	PE150	97.57	92.12	56.12
LQ3_stomco_3A	Tibetan pig	Stomach	38,089,565	11.43	PE150	97.40	91.57	55.88
LQ4_stomco_3A	Tibetan pig	Stomach	40,200,220	12.06	PE150	97.72	92.44	51.97
LQ5_stomco_3A	Tibetan pig	Stomach	40,118,578	12.04	PE150	97.38	91.39	54.08
LQ6_stomco_3A	Tibetan pig	Stomach	39,907,477	11.97	PE150	97.68	92.27	51.52
